# Use of Data-Biased Random Walks on Graphs for the Retrieval of Context-Specific Networks from Genomic Data

**DOI:** 10.1371/journal.pcbi.1000889

**Published:** 2010-08-19

**Authors:** Kakajan Komurov, Michael A. White, Prahlad T. Ram

**Affiliations:** 1 Department of Systems Biology, University of Texas M.D. Anderson Cancer Center, Houston, Texas, United States of America; 2 Department of Cell Biology, University of Texas Southwestern Medical Center, Dallas, Texas, United States of America; University of Illinois at Urbana-Champaign, United States of America

## Abstract

Extracting network-based functional relationships within genomic datasets is an important challenge in the computational analysis of large-scale data. Although many methods, both public and commercial, have been developed, the problem of identifying networks of interactions that are most relevant to the given input data still remains an open issue. Here, we have leveraged the method of random walks on graphs as a powerful platform for scoring network components based on simultaneous assessment of the experimental data as well as local network connectivity. Using this method, NetWalk, we can calculate distribution of Edge Flux values associated with each interaction in the network, which reflects the relevance of interactions based on the experimental data. We show that network-based analyses of genomic data are simpler and more accurate using NetWalk than with some of the currently employed methods. We also present NetWalk analysis of microarray gene expression data from MCF7 cells exposed to different doses of doxorubicin, which reveals a switch-like pattern in the p53 regulated network in cell cycle arrest and apoptosis. Our analyses demonstrate the use of NetWalk as a valuable tool in generating high-confidence hypotheses from high-content genomic data.

## Author Summary

Analysis of high-content genomic data within the context of known networks of interactions of genes can lead to a better understanding of the underlying biological processes. However, finding the networks of interactions that are most relevant to the given data is a challenging task. We present a random walk-based algorithm, NetWalk, which integrates genomic data with networks of interactions between genes to score the relevance of each interaction based on both the data values of the genes as well as their local network connectivity. This results in a distribution of Edge Flux values, which can be used for dynamic reconstruction of user-defined networks. Edge Flux values can be further subjected to statistical analyses such as clustering, allowing for direct numerical comparisons of context-specific networks between different conditions. To test NetWalk performance, we carried out microarray gene expression analysis of MCF7 cells subjected to lethal and sublethal doses of a DNA damaging agent. We compared NetWalk to other network-based analysis methods and found that NetWalk was superior in identifying coherently altered sub-networks from the genomic data. Using NetWalk, we further identified p53-regulated networks that are differentially involved in cell cycle arrest and apoptosis, which we experimentally tested.

## Introduction

An important challenge in the analyses of high throughput datasets is integration of the data with prior knowledge interactions of the measured molecules for the retrieval of most relevant biomolecular networks [1]–[7]. This approach facilitates interpretation of the data within the context of known functional interactions between biological molecules and subsequently leads to high-confidence hypothesis generation. Typically, this procedure would entail identification of genes with highest or lowest data values, which is then followed by identification of associated networks. However, retrieval of most relevant biological networks/pathways associated with the upper or lower end of the data distribution is not a trivial task, mainly because members of a biological pathway do not usually have similar data values (e.g. gene expression change), which necessitates the use of various computational algorithms for finding such networks of genes [1], [2], [4], [5], [8]–[11]. One class of methods for finding relevant networks utilize optimization procedures for finding highest-scoring subnetworks/pathways of genes based on the data values of genes [2], [8]. Although this approach is likely to result in highly relevant networks, it is computationally expensive and inefficient, and is therefore not suitable for routine analyses of functional genomics data in the lab. The most popular of the existing methods of extraction of relevant networks from genomic data, however, usually involve a network building strategy using a pre-defined focus gene set, which is typically a set of genes with most significant data values (e.g. most over-expressed genes) [1], [7]. The network is built by “filling in” other nodes from the network either based on the enrichment of interactions for the focus set (IPA -Ingenuity Pathway Analysis) [1], or based on the analysis of shortest paths between the focus genes (MetaCore) [7], [12]. Both methods aim at identifying genes in the network that are most central to connecting the focus genes to each other. Problems associated with these methods have been outlined previously [7]. However perhaps most importantly, the central genes identified by these methods may have incoherent data values with the focus genes (e.g. the central genes may have reduced expression while the focus genes may have increased expression), as data values of nodes are not accounted for during the network construction process using the seed gene list. This may result in uninformative networks that are not representative of the networks most significantly represented in the genomic data (see Results). In addition, these methods do not account for genes with more subtle data values that collectively may be more important than those with more obvious data values [13]. Although powerful data analysis methods for finding sets of genes with significant, albeit subtle, expression changes have been developed (e.g. GSEA [13], Molecular concept maps[14], GenMAPP[15]), such an approach has not been incorporated into methods for extracting interaction networks that are most highlighted by the data.

In order to overcome these problems, we have employed the method of random walks in graphs for scoring the relevance of interactions in the network to the data. The method of random walks has been well-established for structural analyses of networks, as it can fully account for local as well as global topological structure within the network [16], [17] and it is very useful for identifying most important/central nodes [16]–[18]. Here, instead of working with a pre-defined set of focus genes, we overlay the entire data distribution onto the network, and bias the random walk probabilities based on the data values associated with nodes. This method, NetWalk, generates a distribution of Edge Flux values for each interaction in the network, which then can be used for dynamical network building or further statistical analyses. Here, we describe the concept of NetWalk, demonstrate its usefulness in extracting relevant networks compared to Ingenuity Pathway Analysis, and show the use of NetWalk results in comparative analyses of highlighted networks between different conditions.

We tested NetWalk on experimentally derived genomic data from breast cancer cells treated with different concentrations of doxorubicin, a clinically used chemotherapeutic agent. Using NetWalk, we identify several previously unreported network processes involved in doxorubicin-induced cell death. From these studies we propose that NetWalk is a valuable network based analysis tool that integrates biological high throughput data with prior knowledge networks to define sub-networks of genes that are modulated in a biologically meaningful way. Use of NetWalk will greatly facilitate analysis of genomic data.

## Methods

### Calculating node probabilities using data

Integration of genomic data represented by a vector **w** with the network data of interactions between genes (nodes) is performed by representing each interaction (edge) in the network in the form of a transition probability based on the data values (e.g. mRNA expression change, phenotype score from a genetic screen) of nodes within the immediate neighborhood:
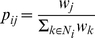
(1)where *p_ij_* is the transition probability from node *i* to node *j*, *w_j_* is the experimental value for node *j*, and *N_i_* is the set of immediate downstream neighbors (undirected edges are considered bidirectional) of node *i*. If there are no downstream nodes of the node *i* (|*N_i_*| = 0), *p_ij_* is set to *p_ij_* = 1/|*n*| for all *j* 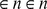, where *n* is the set of all nodes in the network. The relevance score of each node in the network is defined by the probability of its visitation by the random walker, which is a function of both the local network connectivity as well the data values of nodes. So at any step *k* of this “random walk” process, the probability of a node being visited by the random walker is
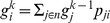
(2)where 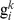 is the probability of node *i* at step *k*, *p_ji_* is the transition probability from node *j* to node *i* and *N* is the set of interacting neighbors of node *i*. This can be represented in a matrix form
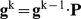
(3)where **g** ^k^ is the vector of probability values for all nodes in the network at step *k*, and **P** is the transition probability matrix of the network. Obviously, since a “walk” can only be performed over adjacent nodes, *p_ij_*>0 only if nodes *i* and *j* directly interact. The expression above can also be written as
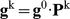
(4)where 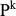 is the transition probability matrix raised to the power *k*, and 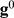 is the initial probability distribution over nodes (all 1/|n|). By the Perron-Frobenius theorem for stochastic matrices, as 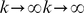 (infinite random walk), the expression above converges to
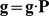
(5)where **g** is the left eigenvector of **P** associated with eigenvalue 1 and contains the final visitation probability values of nodes.

The final visitation probabilities of nodes depend on their data values, data values of their neighbors, as well as the local network connectivity. In order to further bias the random walk towards the input data values, we assigned a small probability *q* that the random walker will return to its starting node. Therefore, the expression for random walk with restart is given by
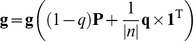
(6)where **q** is a vector of all *q* of length |*n*| and **1** is a vector of all 1: so that the restart probability is uniform among all nodes. However, we bias the restart probabilities to the data values of nodes, so that the random walker is more likely to return to its initial node if the data value of that node is high.
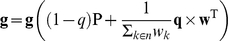
(7)In this way, the probability that the random walker will restart at another node *i* is directly proportional on the data value of node *i*, thereby even more biasing the process of random walk to the biological data.

### Calculating node probabilities for transcription factors

In the case of transcription factor - target gene interactions, these were reversed in the network so that the node values of target genes would contribute to the probabilities of the transcription factors, rather than the other way around. This is because the data values of target genes (i.e. mRNA expression change) are more informative of identifying regulation by transcription factors.

### Calculating edge flux values

To find networks of interactions between genes represented in the data, we scored each interaction in the network by
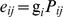
(8)where *e_ij_* is the flux through edge *ij* and represents the score of *importance* of the interaction based on the data.

### Controlling for topological bias in the network

The node visitation frequencies in a random walk directly reflect the relative centralities of nodes in the network, and therefore are highly biased towards the local network topology. Although biasing the random walk to data values skews the visitation frequencies towards the supplied data values, there is still a significantly high correlation with node connectivity values (Figure S1), which suggests that the random walk process is highly biased to the highly connected hubs in the network. Therefore, it is important to control for topological bias in the network that stems either from its scale-free nature or the historical bias of highly studied genes. In order to control for topological biases in the network, we also calculated background visitation frequencies
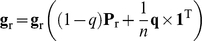
(9)which is the same expression as in equation (7), with the exception that 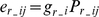. **P_r_** is a transition probability matrix formed by letting *w_i_* = 1 for all *i*. Since **g_r_** is calculated without considering the data values of genes, it contains all the topological bias in the network. Therefore, to obtain relative visitation frequencies of genes (**g′**), we normalize values in **g** by those in **g_r_**,
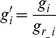
(10)Relative visitation frequency values in **g′** have minimal correlation with node centralities, and have a high correlation with the supplied gene expression measurements (Figure S2), which indicates that relative visitation frequencies of nodes are highly biased towards the data.

Normalization of edge flux values is done by first calculating
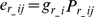
(11)where **e_r_** is the edge score distribution vector calculated by letting *w_i_* = 1 for all *i*. Then, we normalize the data-biased edge flux values to **e_r_** to obtain normalized Edge Flux of interaction *ij* 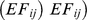


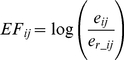
(12)which gives the final normalized score distribution of edges, which reflects edge fluxes of nodes *relative* to what would be expected by topology alone in the given network.

### Data format and missing values

Because of the nature of random walks described above, the input values must be positive, possibly representing ratio of a test versus control sample (e.g. ratio of mRNA expression levels of treated to untreated samples). Missing values in the network are then assigned a value of 1, which represents a *no change* case in ratio values. Accordingly, the values of *s* are centered around 0, with higher values meaning higher probability relative to what would be expected by chance in the given network (i.e. networks of high data value nodes, e.g. increased gene expression), and lower values meaning lower visitation probability (i.e. networks with low data values, e.g. reduced gene expression) (see below).

### Effect of data distribution on Edge Flux values

In order to prevent disproportionate skewing of the node probabilities with extreme outliers in the data, the input data is normalized so that all *w*>k_0.999_ are assigned k_0.999_, where k_0.999_ is the 99.9th percentile value of *w*. Similarly, all *w*<k_0.001_ are assigned k_0.001_. With this procedure, the final normalized visitation frequencies of nodes are highly robust to differences in data distributions and ranges (see Figure S3).

### Network construction

We compiled protein-protein interactions from online databases HPRD [19] BIND [20], HomoMINT [21], Gene [22] and IntAct [23]. For directed interactions, we compiled signaling interactions from KEGG [24], BioCarta (http://pid.nci.nih.gov/) and TRANSPATH [25], as well as through manual curation of the undirected interactions based on published literature. Transcription factor-target interactions were obtained from ORegAnno [26] and TRANSFAC [27] databases. This resulted in a network of 10,473 genes connected by ∼65,000 interactions.

In network-based analyses of genomic data, the analyses and therefore resultant hypotheses are limited by the gene coverage of the network. Therefore, it is crucial that the interaction network has as much gene coverage as possible. Since our main goal of network-based analyses is identification of relevant biological processes, the interactions represented in the network need not be direct physical interactions. For example, a concordant increase in the expression of genes involved in glucose metabolism will not be captured in network-based analyses of direct physical interactions, as metabolic enzymes within the same pathway rarely engage in direct physical interactions (with the exception of multifunctional complexes). Therefore, inclusion of *indirect* functional interactions in the network may help identify relevant biological processes that are not captured by direct interactions (see network plots below). In order to increase the coverage of our network, we added functional similarity interactions between genes, where an interaction means that the genes are involved in similar functional processes, such as a metabolic pathway (e.g. glycolysis) or a specific enzymatic reaction (e.g. oxidation/reduction). Functional similarity interactions were constructed using Gene Ontology (GO) annotations [28] as defined in the Entrez Gene database, and also metabolic pathway annotations in the KEGG database. Any two genes sharing a metabolic pathway annotation (but not signaling pathways as they are already represented in protein-protein interactions) from KEGG were assigned an interaction. In the case of GO annotations, two genes were assigned an interaction if the overlap of their GO annotations was significant compared to the rest of the genes:
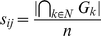
where *s_ij_* is the significance of overlap between genes *i* and *j*; *G_k_* is the set of genes that have the GO term *k*; *N* is the set of GO terms common to genes *i* and *j*, and *n* is the total number of genes. If *s_ij_*<0.001, genes *i* and *j* were assigned an interaction.

Our final network contains 14,506 genes connected by 189,901 interactions. Gene coverage of our network of genes in our doxorubicin dataset is comparable to that in the Ingenuity Pathway Analysis (13,329 in our network versus 13,880 in IPA).

### Microarray analyses

MCF7 cells were grown in DMEM (Invitrogen) supplemented with 10% FBS (Gemini) to near confluency and treated with 1 or 10 µM Doxorubicin (Sigma). Cells were collected at 0, 6, 12 and 24 hours post-treatment. Cell lysis and RNA extraction was done using Mirvana miRNA isolation kit (Ambion) and amplification using Illumina TotalPrep RNA amplification kit (Ambion). Equal amount of RNA from each sample was hybridized to Illumina HT12 BeadChip (Illumina). All procedures were performed exactly as described in the respective manuals. The experiments were repeated in triplicate.

### Analyses with IPA

Networks in IPA were generated using Core analysis with indicated data cutoffs for upregulated genes and using direct interactions with the cutoff for network size to be 70. Highest scoring 5 networks were merged and exported as text files.

### Network plotting

All network plottings were done using the *gplot* function in the *sna* package for R (http://erzuli.ss.uci.edu/R.stuff/).

### Western blotting

Cells were treated as indicated and lysed in a sample lysis buffer (50 mM Hepes, 150 mM NaCl, 1mM EGTA, 10 mM Sodium Pyrophosphate, pH 7.4, 100 nM NaF, 1.5 mM MgCl_2_, 10% glycerol, 1% Triton X-100 plus protease inhibitors; aprotinin, bestatin, leupeptin, E-64, and pepstatin A). Blotting was done using antibodies against p53 (Cell Signaling), p21 (Cell Signaling) and Actin (Sigma). The experiment was done in triplicate.

### Apoptosis assays

FACS: Cells were treated as indicated and after 24 hours trypsinized, fixed with 70% ethanol at −20°C for 10 minutes and resuspended in Propidium Iodide solution. FACS analysis was performed in the Flow Cytometry core facility of M.D. Anderson Cancer Center.

Rhodamine 123 assay: Rhodamine 123 staining was performed as described [29]. Briefly, cells were treated as indicated and after 24 hours, trypsinized, spun down and resuspended in 10 µM Rhodamine 123 (Invitrogen) in PBS for 30 minutes. Cells were washed in PBS and analyzed by FACS for Rhodamine 123 intensity (green).

## Results

### Generating networks with NetWalk

**Figure 1:**
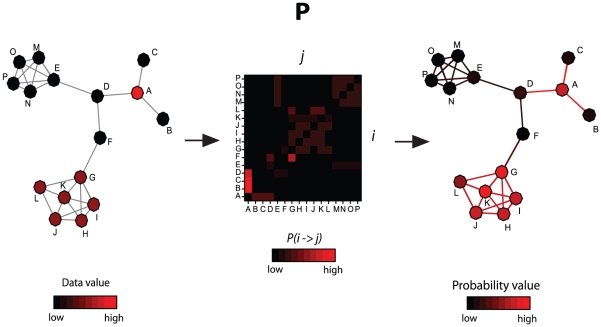
General concept of NetWalk. An imaginary network with artificial experimental data values is shown (e.g. relative gene expression values) on the left. Node A was assigned a value of 5, nodes G, H, I, J, K and L were assigned 2, and all the other nodes were assigned 1. A transition probability matrix **P** was constructed using the input data values and the network, with transition probabilities between adjacent nodes reflecting their data values (colors in the matrix reflect transition probabilities *P(i→j)* according to the color key). Final visitation and flux values reflect the level of coherence between the experimental data of genes and their relative positioning within the network. Note that node colorings in the network on the right reflect relative visitation probabilities of nodes, and line colors of edges reflect the flux values according to the same color scale.

Identifying common biological roles of genes whose expression are altered in a microarray experiment is one of the most frequently used strategies to understand the underlying biological processes and derive hypotheses [6], [13]–[15], [30]. This strategy is also implicit in NetWalk (Figure 1), as node visitation frequency values (hence *EF* values) calculated by NetWalk are based on 1) data values of nodes, 2) data values of their network neighbors and 3) the network connectivity among neighbors. Therefore, a node with a high data value that interacts with other nodes with high data values in the network will receive the highest node visitation and *EF* scores. Similarly, a node with a low data value that interacts with other nodes with low data values in the network will receive the lowest node visitation and *EF* scores.

In order to test the dependency of NetWalk output on the provided data, we performed deletions of portions of data and compared the resultant visitation frequencies to those of the original dataset. Correlation of node visitation frequencies to those of the full dataset closely followed the input data, suggesting that NetWalk output is highly dependent on the supplied data (Figure S4). However, this may also suggest that NetWalk output is mostly independent of the network connectivity. In order to test the dependence of NetWalk output on the network connectivity, we removed parts of the network and performed NetWalk analysis on the perturbed networks. The resultant node visitation frequencies correlate relatively poorly with those of the original network (Figure S5), indicating that the network connectivity substantially contributes to node visitation frequency values. We also performed a similar analysis with random deletions and additions of edges, rather than nodes, in the network, and found a similar dependence of the NetWalk output on the network connectivity (Figure S7). These analyses demonstrate that NetWalk output is highly dependent on both the supplied data as well as the network information.

**Figure 2:**
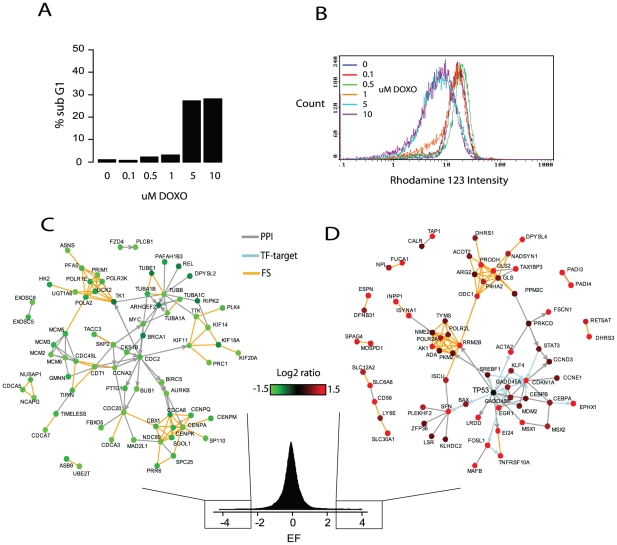
NetWalk analysis of low and high-dose doxorubicin response in MCF7 cells. A) Apoptosis levels in MCF7 cells after 24 hours of stimulation with indicated doses of doxorubicin as measured by FACS analysis of DNA content (see Methods). B) FACS analysis of viable cells as indicated by loss of Rhodamine 123 staining(see Methods). C–D) Plots of interactions with lowest(B) and highest (C) EF values in samples treated with 1 µM doxorubicin for 24 hours relative to control. Nodes are colored according to their gene expression change relative to control according to the color key. Edge coloring reflects type of interaction, PPI: protein-protein interaction, TF-target: gene regulation, FS: functional similarity. The distribution plot of all EF values is shows at the bottom.

To demonstrate the use of NetWalk in the extraction of relevant networks out of microarray gene expression data, we studied gene expression profiles of MCF7 cells subjected to sub-lethal and lethal doses of doxorubicin. We performed microarray gene expression analysis of MCF7 cells before and after treatment with 1 or 10 µM doxorubicin for 6, 12 and 24 hours. In these cells, 1 µM doxorubicin causes a cell cycle arrest in S-phase, while a 10 µM dose induces cell death (Figure 2A–B). A NetWalk analysis of the ratio values (treated/untreated) for 1 µM treatment was performed using *q* = 0.01 (see Methods). The resulting distribution of edge flux values, and plots of edges with 100 highest and lowest *EF* values can be seen in Figure 2C–D. *EF* values are strictly biased towards the data, as the high and low-end networks are entirely composed of genes with, respectively, increased and reduced expression levels. In the Figure 2D, interactions in the cluster made of GLS, GLS2, P4HA2, ODC1 and PRODH genes (arginine and proline metabolism) have the highest *EF* scores due to both their high data values and tight interconnections with each other. Similarly, in the low-score network in Figure 2C, interactions in the cluster containing NDC80, CENPK, CBX1, CENPA and SGOL1 (centriole components) have the lowest *EF* scores. Nodes with moderate values that are in close proximity to other high value nodes within a tightly connected neighborhood will also get high scores, as is seen with TP53 in Figure 2B.

In order to demonstrate that the p53 network extracted by NetWalk is not an artifact of highly connected subnetworks, we performed a NetWalk analysis of baseline expression profile of MCF7 cells relative to other breast cancer cells as reported by Neve *et al* [31]. The most significantly upregulated networks in MCF7 cells relative to the rest of 53 breast cancer cells are those involved in the Estrogen Receptor signaling (Figure S6), a well-characterized dominant pathway in the estrogen receptor positive MCF7 cells. This analysis shows that NetWalk output does indeed reflect accurate quantification of highly biologically relevant networks based on the supplied data.

### *EF* scores are highly coherent with data values

**Figure 3:**
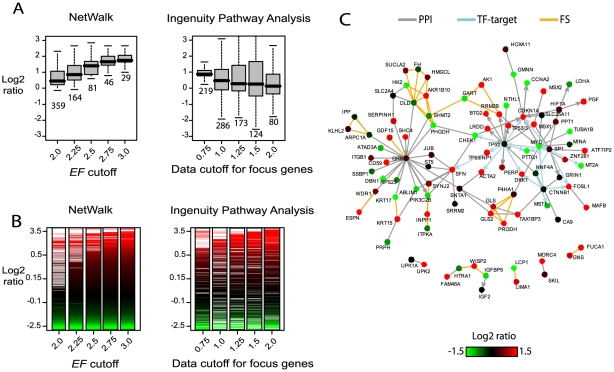
Comparison of coherence of node values in highest scoring networks. A) Boxplots of gene expression change values (1 µM DOX, 24 hours relative to control) of nodes in networks generated by different cutoffs of EF values, or in networks generated by Ingenuity Pathway Analysis software using different gene expression value cutoffs for the focus gene set (see Methods). B) Heatmaps showing position of genes in the networks in A in the whole data distribution. Positions of genes in the respective networks are indicated by a white line. C) A network of nodes generated by Ingenuity Pathway Analysis software with focus gene set using 1.5 as cutoff. Since original network plots in IPA lack node colorings for intermediate genes (non-focus genes), we extracted all nodes in the IPA-generated network and re-plotted them using our network, where we colored all nodes by their gene expression change.

Contrary to the seed-based network building methods, NetWalk works with the whole data distribution and so does not require assignment of pre-defined cutoffs or focus gene sets. NetWalk procedure simply translates the gene-centric data values to corresponding interaction scores based on the coherence of the gene values with those in the local network neighborhood as well as the local interaction pattern in the network. Therefore, the results can be viewed at any user defined cutoff value for flexible generation of networks with highly coherent node values. The distribution of input node values and sample networks with different *EF* cutoffs shows that the node values within networks are highly coherent across a wide range of *EF* score cutoffs, which allows for high-confidence hypothesis generation about activated and inactivated network processes in response to DNA damage (Figure 3A–B). In comparison, the distribution of data values of nodes in the networks generated by Ingenuity Pathway Analysis, which takes a focus gene list as input to build relevant networks, includes nodes with incoherent data values (see Figure 3A–C), which reduces confidence in the relevance of the generated networks to the data. The network of 124 genes retrieved by IPA using a cutoff of >1.5 (60 focus genes) contains many genes with reduced expression values (Figure 3C), which were included in the network by the virtue of their connectivities but not data values. Consequently, the resulting network is not entirely representative of upregulated network processes in response to doxorubicin. Moreover, none of the networks identified by IPA contain all the genes involved in arginine-proline metabolism (compare Figures 2D and 3C) or any genes involved in the nucleotide metabolism that were retrieved by NetWalk (see cluster in Figure 2D containing RRM2B, AK1, POLR2A and NME2; compare with Figure 3C), demonstrating inability of seed-based methods to identify subnetworks with more subtle yet coherent gene expression values.

### Statistical analyses using NetWalk output to elucidate p53-mediated response to DNA damage

**Figure 4:**
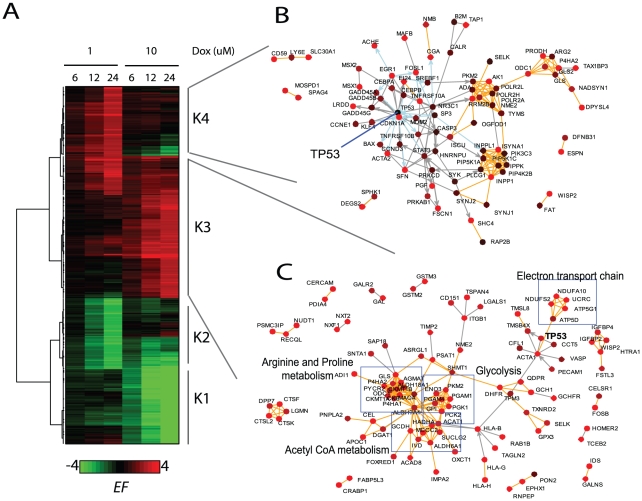
Clustering analysis of EF values in each condition. A) Heatmap of highest and lowest EF values in each condition. Clustering was done using Ward's method in R. B–C) Networks corresponding to K3 (B) and K4 (C). Node colorings are according to 24h of 1 and 10 µM DOX treatments, respectively. Edge colorings are as in Figure 2C.

**Figure 5:**
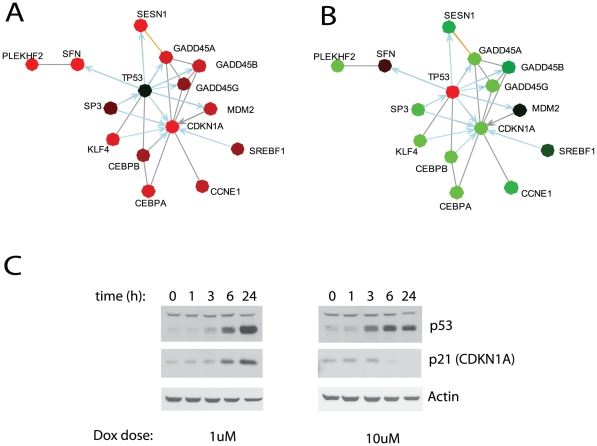
p53-target cell cycle regulatory genes are specifically repressed during apoptosis. A–B) Network plot of interactions in K3 (see Figure 4) related to cell cycle regulation. Nodes colored according to gene expression changes at 10 (A) or 1 µM (B) doxorubicin treatment. C) Western blots of p53, p21 (CDKN1A gene product) protein levels over a time course after 1 and 10µM doxorubicin treatment. Actin levels shown as control.

As stated earlier, an important feature of NetWalk is that the result is not a single or a collection of static networks, but a whole distribution of numerical edge scores. In addition to their use for dynamical network construction of different sizes based on the user preference, these can be further subjected to standard statistical tests for a more detailed analysis. The heatmap of interactions with highest and lowest *EF* scores in each condition in our microarray dataset is shown in Figure 4A. As opposed to clustering with traditional heatmaps of gene expression values where cluster membership of genes is exclusive, here, a gene can appear in several different clusters but all with different interactions. So, analysis of expression with *EF* scores enables studying specific functions (i.e. interactions) of genes rather than their individual expression values. The heatmap shows that the activation and/or inactivation of several networks is specific to low- or high-dose doxorubicin treatment. The cluster K3, for example, is activated in response to high-dose doxorubicin, while K4 is more specifically activated in response low-dose doxorubicin. A plot of interactions in K3 reveals several metabolic pathways specifically activated in the high-dose treatment, including glycolysis, acetyl coenzyme A synthesis, arginine/proline metabolism and the mitochondrial electron transport chain (Figure 4C). There is also a p53-centered subnetwork containing several previously identified p53 target genes. The plot of interactions in K4 shows an extensive p53-centered network composed mostly of cell cycle regulatory proteins (e.g. CDKN1A (p21CIP) and several GADD45 genes) (Figure 4B). Interestingly, although p53 appears in both K3 and K4, its functions seem to be completely different in the low and high dose treatments. In response to low-dose doxorubicin, p53 is involved in the activation of cell cycle regulatory proteins, while under high-dose, it activates other targets, such as TMSB4X. Moreover, p53-target genes in cell cycle regulation in K3 are inactivated in high-dose doxorubicin (Figure 5A–B), which we confirmed by western blotting (Figure 5C), suggesting that p53 may act as a transcriptional activator of these genes during cell cycle arrest but as a repressor during apoptosis. This trend suggests not only that p53 may engage different targets during cell cycle arrest and apoptosis, but also shows dual behavior of p53 under these conditions. In addition, this analysis shows that energy and amino acid metabolisms may play an important role in doxorubicin-induced cell death. Here, clustering analysis using NetWalk results facilitated comparison of networks, rather than genes, between different conditions, leading to the identification of differential activities of p53 under low and high-dose doxorubicin treatment.

## Discussion

### NetWalk algorithm

Analyses of high content data within the context of biological interactions allow for high confidence hypothesis generation about mechanisms involved in the studied process. While some work has been done on inferring novel causal interactions out of data [32]–[34], the most popular method is integration of data with prior knowledge on interactions to extract most relevant networks highlighted by the data. Most of the methods for extracting relevant networks rely on finding genes in the network that are most central to connecting the genes of interest identified from the data. The random walk process in NetWalk also scores most central genes in the network. However, rather than working on a small set of focus genes, NetWalk scores centralities of all genes in the network based on the whole data distribution. This is achieved by biasing the random walk transition probabilities between genes to their corresponding data values, which allows for higher visitation probabilities of nodes with high data values and lower probabilities of nodes with low data values. Since visitation probabilities of nodes in a random walk are also dependent on the visitation probabilities of their network neighbors, nodes with relatively moderate data values associated with those with higher values have the potential of high visitation by the random walk. Therefore, NetWalk scores nodes based on their data values, data values of their neighbors and local network connectivity.

Unlike most of the existing methods for network extraction, which typically give a set of networks as outputs [1], [9], NetWalk gives a distribution of *EF* values that allows for flexibility in network construction using different *EF* cutoffs. In addition, *EF* scores can be subjected to further statistical tests for comparative studies, allowing for network-based comparisons of multiple conditions.

Another important feature of NetWalk is its computational efficiency. We implemented a sparse matrix representation and multiplication, which allows for NetWalk to be run on a standard PC equipped with 1 gigabytes of memory. In our case (PC with Intel Xeon Quad processor), NetWalk run of a single dataset in our network (14,506 nodes and ∼190,000 interactions) took about 2–3 seconds.

NetWalk analysis of the experimental data revealed a significant activation of networks involved in energy metabolism, including the glycolytic and mitochondrial electron transport chain components. At least one member of the electron transport chain, SCO2A, has been previously shown to be a p53 target [35], suggesting that some, if not most, of the metabolic genes activated in response to 10 uM doxorubicin may be p53 target genes. A specific and extensive activation of the energy metabolism during p53-mediated apoptosis has not been previously reported, and therefore it is a novel finding facilitated by NetWalk analysis. Network analysis of experimental data using NetWalk revealed dual behavior of p53 under sublethal and lethal doses of DNA damage. In response to sublethal doses of DNA damaging agents, p53 activates a cell cycle arrest program centered around CDK inhibitors p21 (CDKN1A) and GADD45, as well as several pro-apoptotic genes, such as BAX and APAF1. However under lethal doses, p53 represses the cell cycle arrest machinery and activates an entirely different program. Use of NetWalk analysis allows network based analysis of genomic data as well as high confidence hypothesis generation and is a valuable tool in post-genomic anlaysis.

## Supporting Information

Figure S1 Correlation of node visitation frequencies with node connectivities (left) and original data values (right) before normalization for network topology (see Text). R2 values show squared Spearman's rank correlation coefficients.
(0.08 MB PDF)

Figure S2 Same as in Figure S1, but after normalization for network topological bias (see Text).
(0.09 MB PDF)

Figure S3Effect of data range on NetWalk output. Original mRNA expression changes in response to 1uM doxorubicin (ratio) were log2-transformed (di), and then transformed back by taking exponential with different expansion factors f, σ_i = f
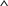
(d_i ) where σ_i is the transformed value of gene i, di is the log2-transformed original ratio value of gene i and f is the expansion factor. Distributions of the transformed data with different expansion factors are shown in A. Numbers above each distribution chart shows the expansion factor. Expansion factor of 2 corresponds to the original distribution. B) Correlation of visitation frequencies corresponding to each transformed dataset with the original visitation frequency values (i.e. f = 2). C) Correlation of visitation frequency values for each expansion factor with the supplied transformed data values. D–E) Highest scoring interactions calculated using transformed datasets with expansion factor D) 1.25 and E) 5. Note that the two networks are highly similar ∼ 95% same node composition).(0.23 MB PDF)

Figure S4 Effect of data deletions on NetWalk output. Portions of data were deleted and node visitation frequencies were calculated by NetWalk. Shown are the correlations of each deletion with the original node visitation frequency values (i.e. 0% deletion).
(0.03 MB PDF)

Figure S5 Effect of network deletions on NetWalk output. A network corresponding to 690 nodes (highest scoring interactions in 1uM doxorubicin dataset) was selected and nodes were deleted at random. Correlation of resulting node visitation frequency values with the original unperturbed network of 690 nodes is shown (black). In addition, corresponding correlations with the node degrees in each networks are also shown. Note that although total number of interactions are relatively similar in each deletion, the NetWalk output changes substantially due to changes in the local network connectivities.
(0.07 MB PDF)

Figure S6 Highest scoring networks corresponding to estrogen receptor positive MCF7 cells relative to 58 other breast cancer cell lines. ESR1 (estrogen receptor gene) is highlighted.
(0.26 MB PDF)

Figure S7 Effect of edge perturbations on NetWalk output. A random network corresponding to 755 nodes was selected out of the whole network (3721 interactions). A) Edges were deleted at random and correlation of the resultant node visitation frequencies were compared to that of unperturbed network. B) To the network in A where 50% of all edges were removed, we added random interactions between random pairs of nodes and compared the resultant NetWalk output with the initial NetWalk output at 50% deleted network.
(0.09 MB JPG)
