# Supplementary figures and images for: Use of Data-Biased Random Walks on Graphs for the Retrieval of Context-Specific Networks from Genomic Data
Source: PLoS Comput Biol. 2010 Aug 19;6(8):e1000889. doi: 10.1371/journal.pcbi.1000889 (PMC2924243; doi:10.1371/journal.pcbi.1000889)

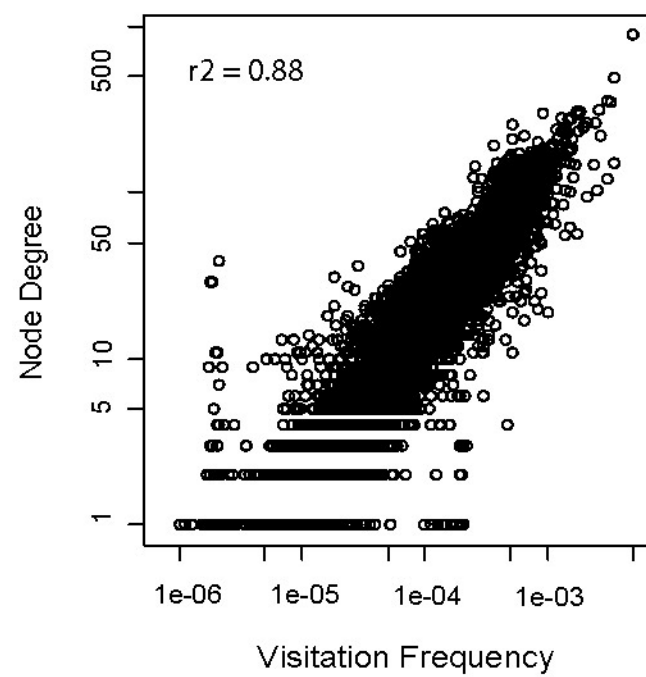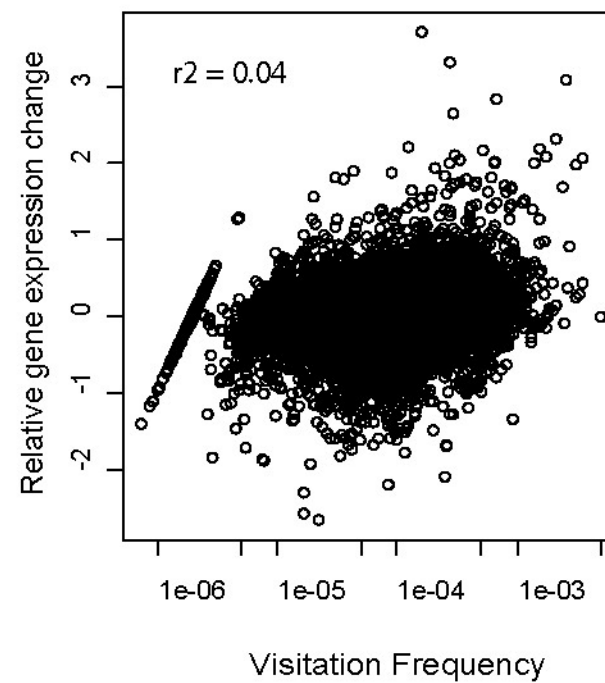

Supplement: Figure S1 — Correlation of node visitation frequencies with node connectivities (left) and original data values (right) before normalization for network topology (see Text). R2 values show squared Spearman's rank correlation coefficients. (0.08 MB PDF) [file pcbi.1000889.s001.pdf]

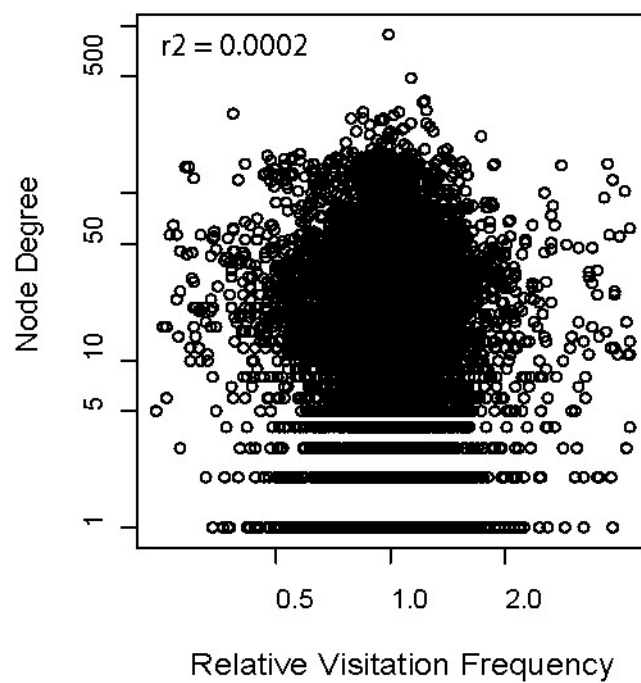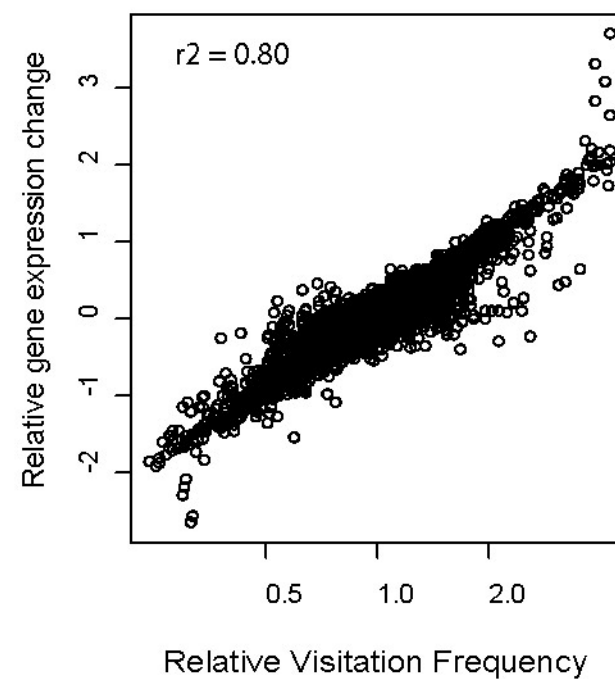

Supplement: Figure S2 — Same as in Figure S1, but after normalization for network topological bias (see Text). (0.09 MB PDF) [file pcbi.1000889.s002.pdf]

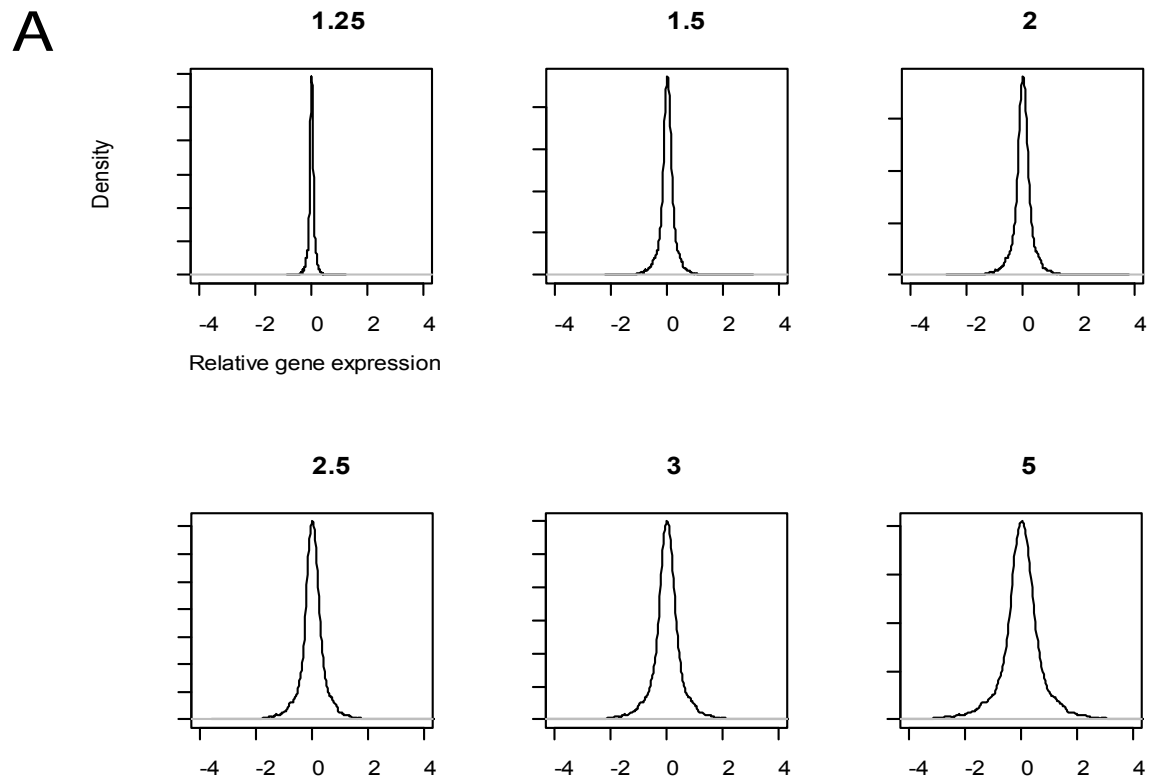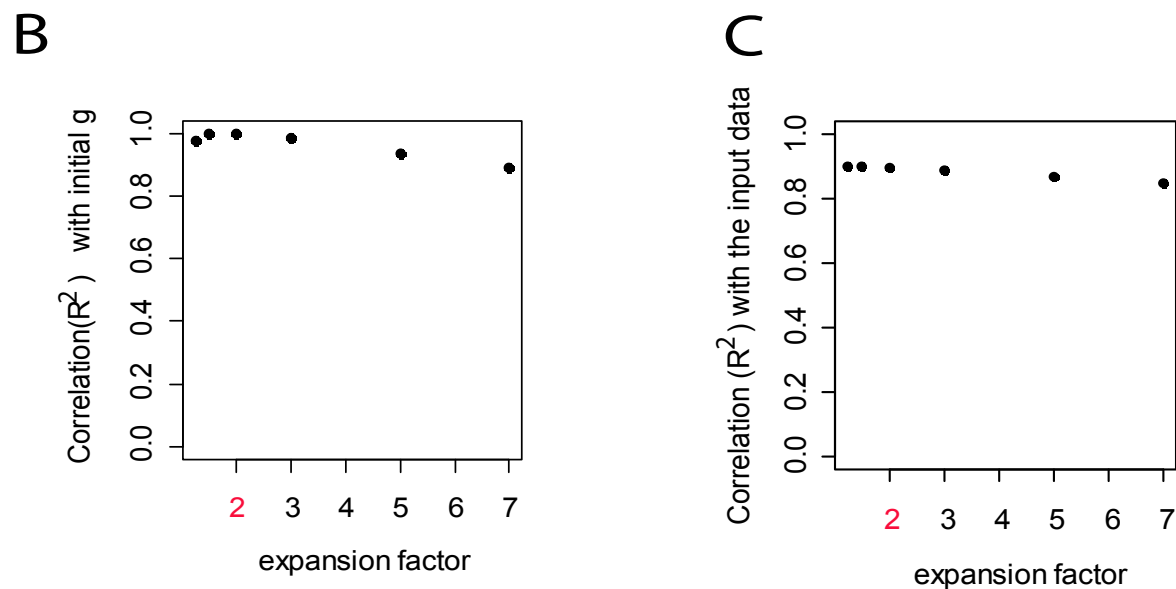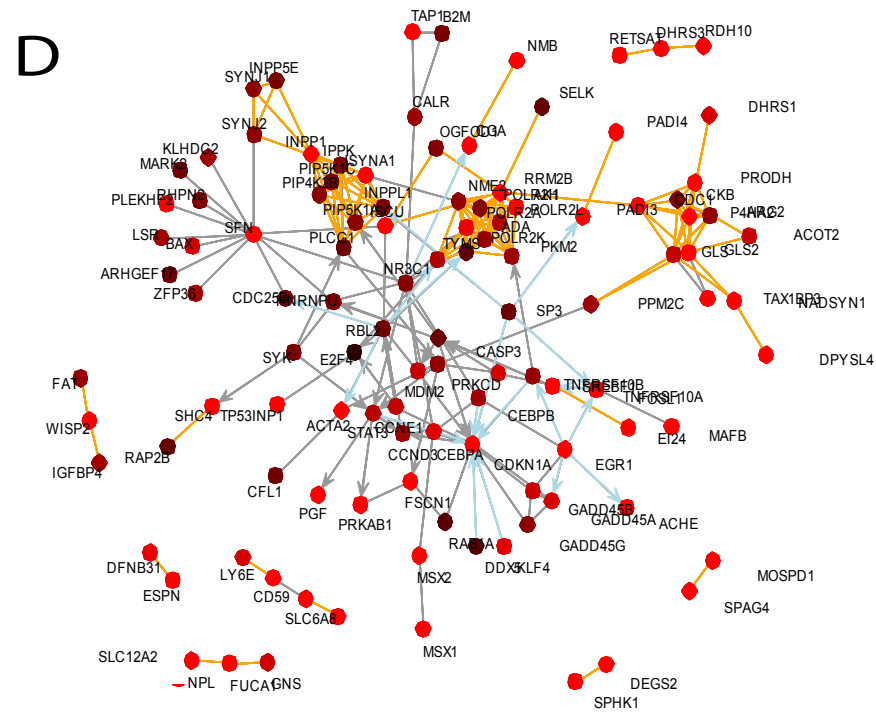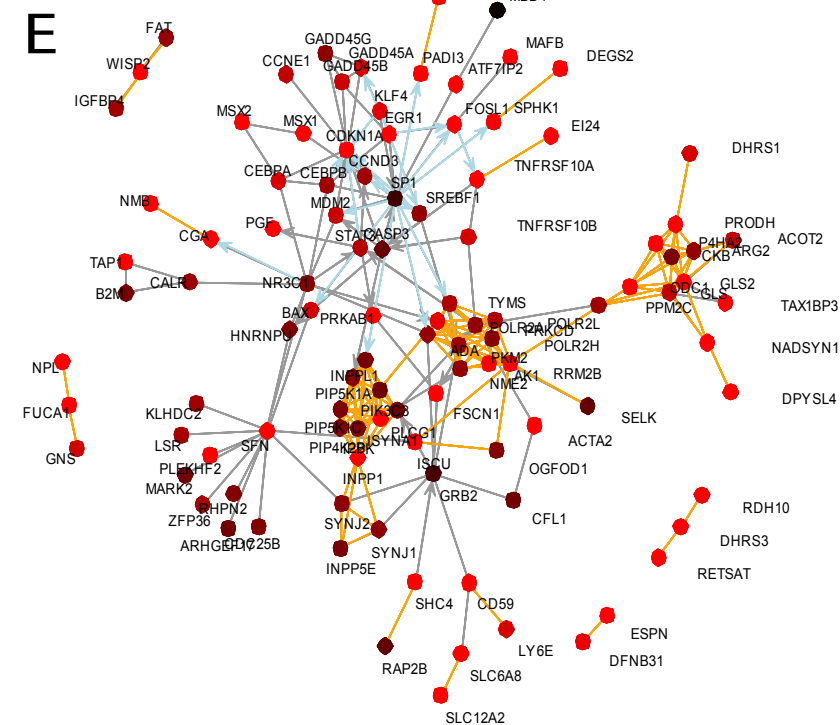

Supplement: Figure S3 — Effect of data range on NetWalk output. Original mRNA expression changes in response to 1uM doxorubicin (ratio) were log2-transformed (di), and then transformed back by taking exponential with different expansion factors f, σ_i = f(d_i ) where σ_i is the transformed value of gene i, di is the log2-transformed original ratio value of gene i and f is the expansion factor. Distributions of the transformed data with different expansion factors are shown in A. Numbers above each distribution chart shows the expansion factor. Expansion factor of 2 corresponds to the original distribution. B) Correlation of visitation frequencies corresponding to each transformed dataset with the original visitation frequency values (i.e. f = 2). C) Correlation of visitation frequency values for each expansion factor with the supplied transformed data values. D–E) Highest scoring interactions calculated using transformed datasets with expansion factor D) 1.25 and E) 5. Note that the two networks are highly similar ∼ 95% same node composition). (0.23 MB PDF) [file pcbi.1000889.s003.pdf]

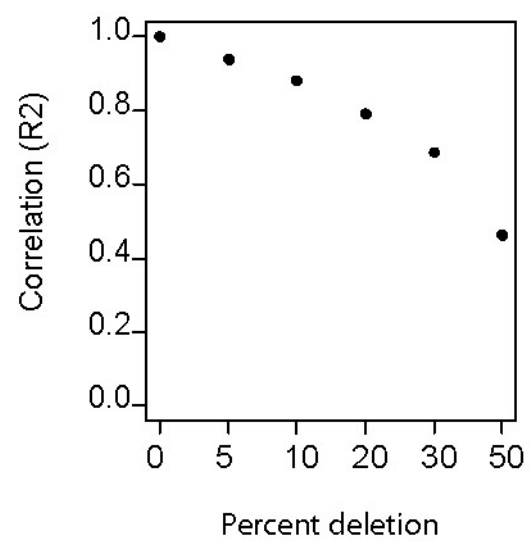

Supplement: Figure S4 — Effect of data deletions on NetWalk output. Portions of data were deleted and node visitation frequencies were calculated by NetWalk. Shown are the correlations of each deletion with the original node visitation frequency values (i.e. 0% deletion). (0.03 MB PDF) [file pcbi.1000889.s004.pdf]

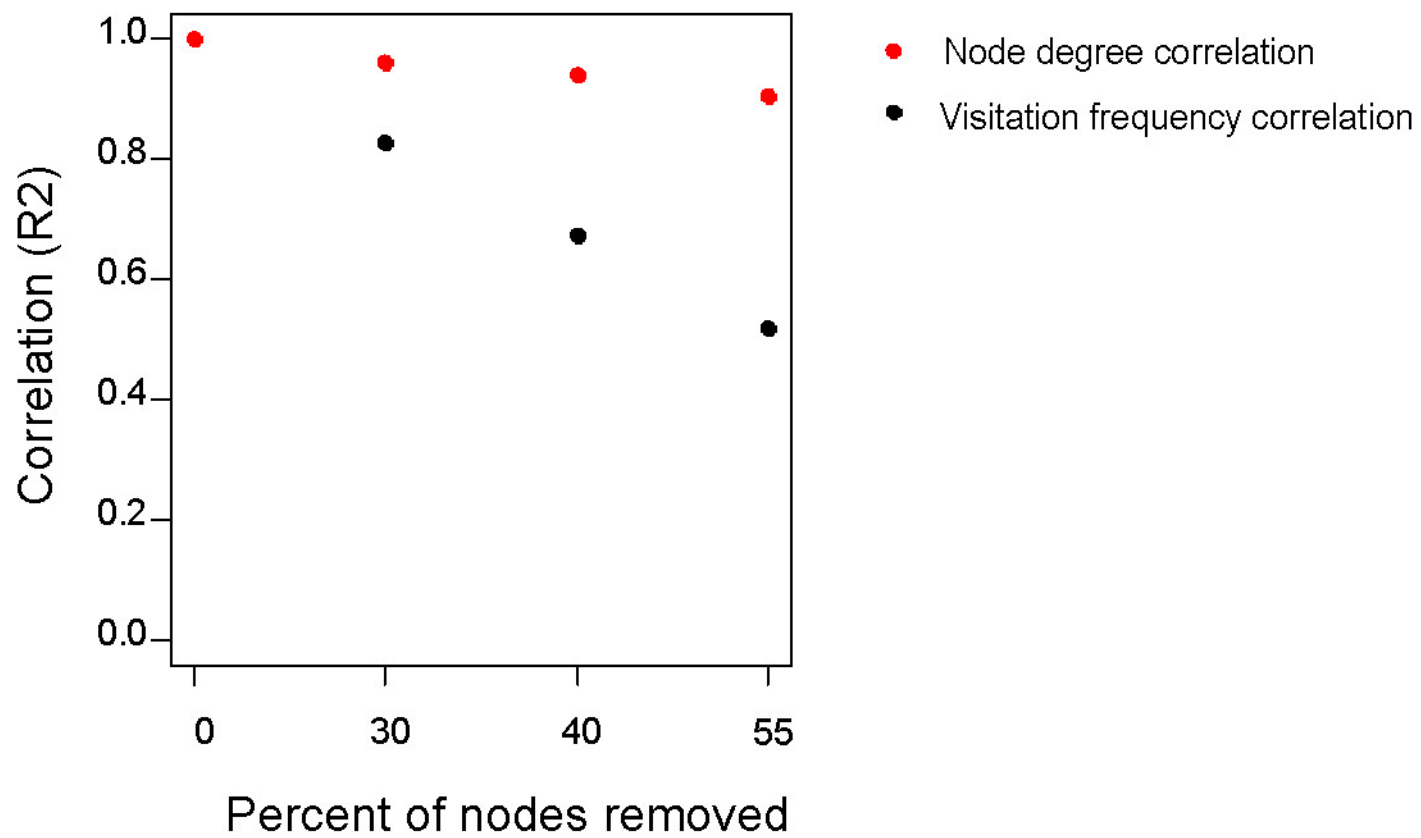

Supplement: Figure S5 — Effect of network deletions on NetWalk output. A network corresponding to 690 nodes (highest scoring interactions in 1uM doxorubicin dataset) was selected and nodes were deleted at random. Correlation of resulting node visitation frequency values with the original unperturbed network of 690 nodes is shown (black). In addition, corresponding correlations with the node degrees in each networks are also shown. Note that although total number of interactions are relatively similar in each deletion, the NetWalk output changes substantially due to changes in the local network connectivities. (0.07 MB PDF) [file pcbi.1000889.s005.pdf]

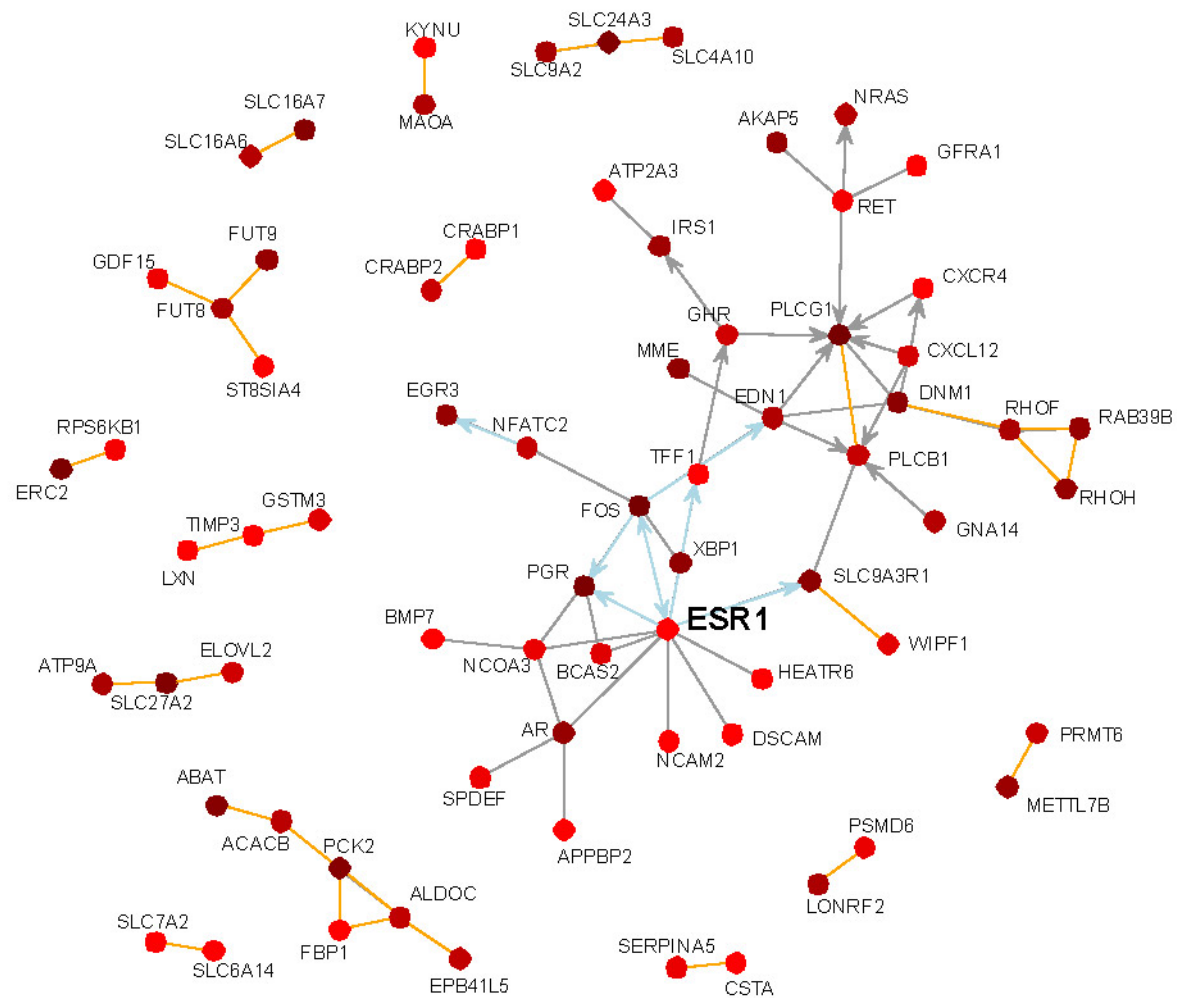

Supplement: Figure S6 — Highest scoring networks corresponding to estrogen receptor positive MCF7 cells relative to 58 other breast cancer cell lines. ESR1 (estrogen receptor gene) is highlighted. (0.26 MB PDF) [file pcbi.1000889.s006.pdf]

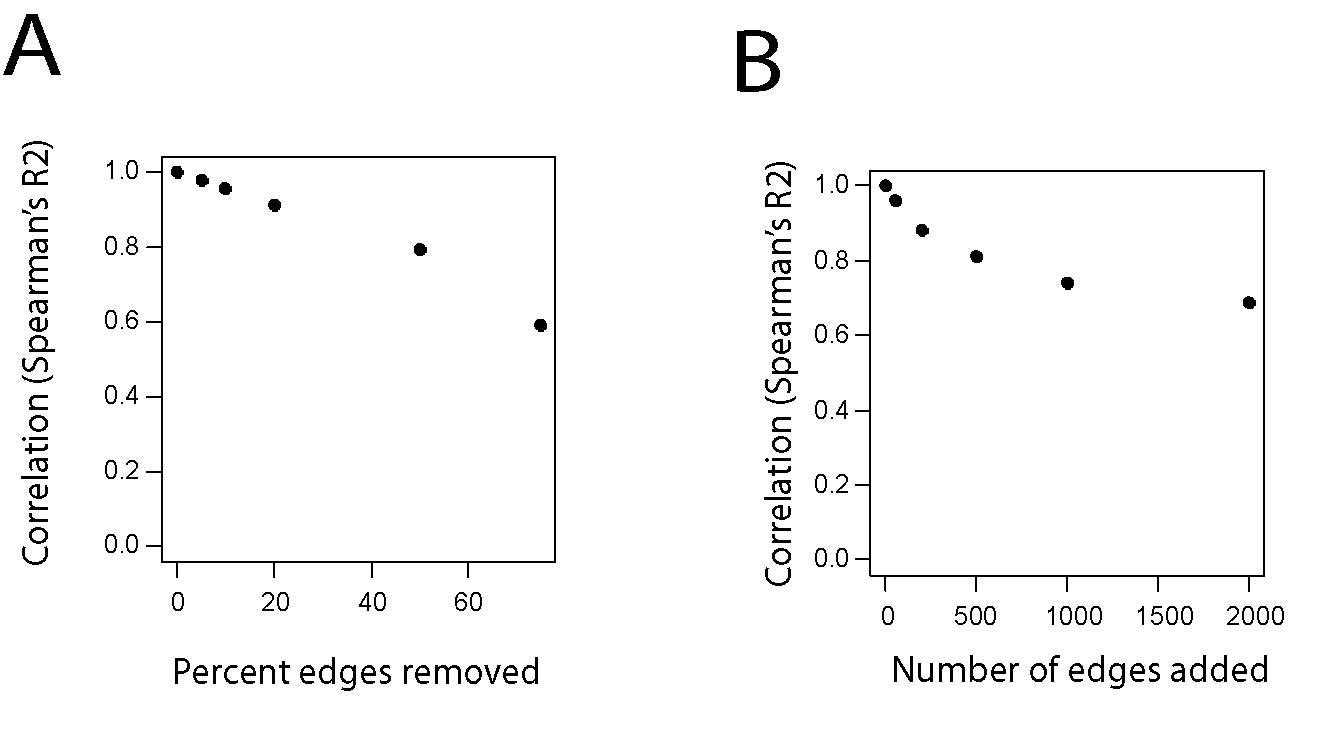

Supplement: Figure S7 — Effect of edge perturbations on NetWalk output. A random network corresponding to 755 nodes was selected out of the whole network (3721 interactions). A) Edges were deleted at random and correlation of the resultant node visitation frequencies were compared to that of unperturbed network. B) To the network in A where 50% of all edges were removed, we added random interactions between random pairs of nodes and compared the resultant NetWalk output with the initial NetWalk output at 50% deleted network. (0.09 MB JPG) [file pcbi.1000889.s007.jpg]
